# Novel semi-quantitative risk model based on AHP: A case study of US driving risks

**DOI:** 10.1016/j.heliyon.2023.e20685

**Published:** 2023-10-05

**Authors:** Cinzia Carrodano

**Affiliations:** University of Geneva, Geneva School of Economics and Management, Information Science Institute, Switzerland

**Keywords:** Risk assessment, Analytical hierarchy process, Driving risk factors

## Abstract

Road safety is a priority, worldwide. The European Commission aims to reduce fatalities by 2030. The same goal was set for the US. These goals stem from the World Health Organization's (WHO's) broader global context, which has distinctly emphasized a substantial reduction in road traffic injuries. Although different risk factors were observed in different geographical locations, the major risk factors for all locations were similar. They involve influencing human behavior, such as speeding or driving. Several methods have been used to better understand and extract risk factors. However, the complexity of road traffic implies the need for a multi-criteria method. As a result, the analytical hierarchy process (AHP) has emerged as a potential method for this type of risk. The AHP is commonly associated with the use of qualitative methods such as surveys. We propose a novel semi-quantitative multi-criteria risk model (SMCRisk) based on the AHP, deployed in a quantitative and partially qualitative manner by adding a severity factor. The multi-level framework differentiates between the driver's behavior and the driver's state. Our method results correspond to a real situation and confirm that driver behavior and state are major risk factors. In future, this method will lay the foundation for integrating a fully quantitative method by considering the potential use of data originating directly from the IoT, which is a part of our research on holistic risk assessment.

## 1 Introduction

Road safety remains a crucial area of study in Europe owing to the persistently high number of injuries and fatalities. By 2030, the European Commission aims to substantially reduce the prevalence of road-related accidents *(*https://transport.ec.europa.eu/news/european-commission-welcomes-launch-global-plan-un-decade-action-road-safety-2021-2030-2021-10-28_en*).*

**Fig. 1:**
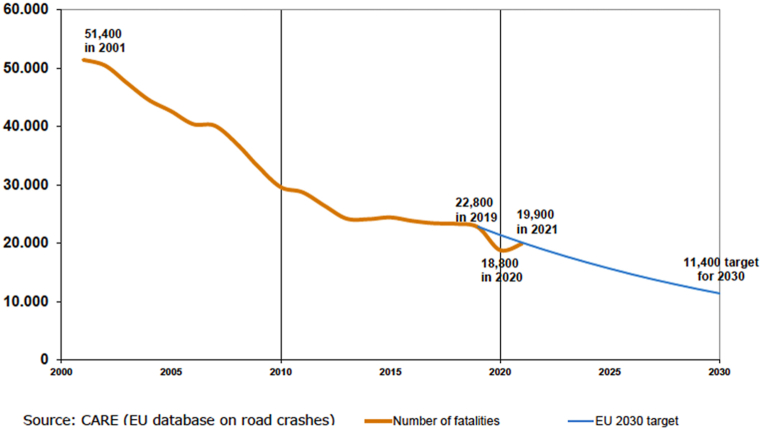
Number of fatal crashes in E.U. (Source: CARE, EU database on the road crashes 2021).

In 2021, Europe recorded 19,900 fatal crashes, which is a 5.9 % increase compared to those recorded in 2020 (Fig. 1). The influence of COVID-related measures, such as diminished vehicle usage, may also be observed, which could explain the slight reduction in accidents in 2020. Although the general trend has been decreasing since the 2000s, this decline has become less pronounced over the last decade.

The notable decline observed before 2015 can be attributed to significant improvements in vehicle technology, such as anti-lock brake systems (ABS) and electronic stability programs (ESP), along with advancements in road infrastructure. Moreover, the introduction of more rigorous disciplinary actions, such as enforced speed limits and penalties for legal violations, has contributed to the decline. Despite these improvements, fatal accident rates continue to be relatively high, and the decrease is marginal. Numerous assessments of traffic risks emphasize driver behaviors, such as driving under the influence of alcohol, the impact of fatigue, and erroneous risk evaluations, such as driving at excessive speeds.

Considering a broader global perspective, the World Health Organization (WHO) has set medium-term goals to decrease road accidents. In their June 2022 analysis *(*https://www.who.int/news-room/fact-sheets/detail/road-traffic-injuries*),* the WHO reported that approximately 1.3 million people die annually from road traffic accidents. The primary risk factors include speeding, driving under the influence of alcohol or other psychoactive substances, not using safety equipment such as seatbelts, and distracted driving. The National Highway Traffic Safety Administration (NHTSA) also identified similar hazardous behaviors on US roads. Although there are some regional differences in the risk factors, the primary ones are predominantly centered on human behavior (https://www.nhtsa.gov/).

Many studies have attempted to understand human errors and risky behaviors by examining factors such as risk perception [[1], [2], [3]], driving styles [4,5], impact of driving experience [[6], [7], [8]], and individual personalities [9,12,13,10,11].

Road traffic safety requires a diverse array of information to identify and comprehend the risk factors.

Multicriteria decision-making (MCDM) methods allow for complex weighting problems such as road traffic safety. There are several methods, and the most well-known are subdivided into subjective and objective classifications [14,15]. The main difference between objective and subjective methods is their structure [14]. Subjective methods are based on the intuition and perception of decision-makers, whereas objective methods are devoid of human opinions. The entropy weight method [16] and criteria importance through the inter-criteria correlation (CRITIC) method [17] are well-known objective methods in MCDM. Subjective methods include several major models, such as the analytic hierarchy process (AHP) method [18], best worst method (BWM) [19], full consistency method (FUCOM) [15], level-based weight assessment (LBWA) method [20], and defining interrelationships between ranked criteria (DIBR) method [21].

This study presents a novel subjective MCDM method called the semi-quantitative multi-criteria risk model (SMCRisk). The innovative aspects of this model are the integration of a severity level using a severity cost factor in the weighting and the pairwise comparison matrix developed considering a quantitative method alongside a qualitative one. This method allows for less subjectivity in determining the weighting process and the intensity of the risk factor for road safety applications.

Hereafter, we discuss some advantages and disadvantages of subjective methods, as well as the contribution of SMCRisk. The widely used AHP, introduced by Saaty, is based on pairwise comparison matrices (PCMs). Razaei [19] introduced the PCM method by Thurstone [22]. This implies structuring the manner in which the decision is made by experts showing relative preferences. The main advantage of the PCM is its simplicity; however, the PCM method suffers from a recurring lack of consistency [19,23]. Razaei proposed the BWM method to address this issue. The BWM requires defining one best and one worst criterion within a set of criteria. Both methods are based on experts subjective estimation, which implies inconsistency and therefore reduces the reliability of the results for a large number of criteria. To address this issue, several models, such as FUCOM, LBWA, and DIBR, have been developed that allow for a more rational method with a higher consistency rate.

Based on the pairwise comparison of criteria and the reduction in the number of comparisons, which implies a higher consistency rate, the major characteristic of FUCOM is to lower the decision maker's subjectivity [15]. Unlike the AHP, which only allows a maximum of nine criteria to be compared, FUCOM allows for the comparison of a large number of criteria. With a small number of criteria comparisons, LBWA was developed in 2019 by Zizovic and Pamucar [20] to address complex MCDM, considering that the AHP and BWM methods became too complex in this case. Another method of rational reasoning and objectivity in alternative evaluations is the DIBR method [21]. The limitations of the AHP and BWM related to a maximum ratio of 9:1 were eliminated using DIBR. This method was used along with the fuzzy Dombi CoCoSo method in a sustainable scenario to improve a city's urban mobility system, where the complexity of the mathematical algorithm was mentioned as a limitation [21].

The challenges for road safety analysis using MCDM methods include defining not only an objective and simple mathematical method but also considering the concept of risk intensity or severity level. Our model integrates risk severity levels into the weighting calculation. In this study, we present the application of the SMCRisk to a real-world case study of driving risks. This model was designed for risk analysis using the MCDM method, which includes not only road risks but also other safety fields.

The AHP offers a fitting solution for modeling complex problems with its hierarchical structure [18]. A complex decision-making problem can be decomposed into a hierarchical structure using this structuring capability. This is particularly useful for driving-risk analysis with multiple layers of criteria and sub-criteria. AHP is also one of the most common and widely used MCDM methods [24].

According to the literature review, the AHP is frequently used either independently or in conjunction with other techniques. Some recent examples include the model proposed by Sivaprakasam and Angamuthu [25], which used generalized Z-fuzzy soft β-covering-based rough matrices based on the AHP method and used it for the recruitment of the best candidate for an assistant professor job. Jagtap and Karande [26] developed the mFS ELECTRE-I combined with an improved Simos and AHP weight method used in the industry sector. Carra et al. [27] proposed a combined AHP and ELECTRE method for comparative cycling path selection. When used independently, the AHP method may be used to select the wastewater treatment technology [28].

The AHP is widely used for risk analysis across various sectors, including industrial construction projects [29], the textile industry [30], epidemic diseases [31], and road safety.

Kaewfak et al. [32] conducted a multimodal freight transportation risk analysis in 2020 using fuzzy AHP and data envelopment analysis (DEA) methods. Moslem et al. [24] performed a more specific analysis of driver behavior factors related to road safety using criteria such as lapses, violations, and errors and combined the AHP with the BWM. In 2021, Farooq et al. [33] focused on frequent lane changes for road safety by using the AHP-BWM technique.

The aforementioned road risk behavioral studies using AHP-based methods used qualitative methods, such as questionnaire surveys, to develop their models. Our study differs in terms of its methodology and modeling. Nonetheless, quantifying driving risk based exclusively on expert opinions presents challenges. We propose incorporating existing empirical data, thereby adding an objective dimension to risk comparison.

According to Kaplan and Garrick [34], risk can be defined using three questions.

(1) “What can happen (i.e. What can go wrong)?
(2) How likely is it that that will happen?
(3) *If it does happen, what are the consequences?*”

While this interpretation of risk provides a wide-ranging comprehension, it is not the only definition. Aven et al. [35] compiled various risk definitions, emphasizing the multifaceted nature of risk and the absence of consensus in formulating a single, unified interpretation. We will use the definition provided by Aven and Renn [36], which highlights the uncertainty and severity of consequences: “*Risk is uncertainty about and severity of the consequences (or outcomes) of an activity with respect to something that humans value*.” Driving risks include the potential consequences and impacts of an event and may vary from minor to substantial. The mathematical foundations of AHP can be used to evaluate and prioritize various factors, considering the uncertainty and severity of their potential consequences.

Our method integrates quantitative and qualitative aspects. Instead of using a questionnaire, we used an empirical statistical method. We provide the severity intensity for the risk assessment by integrating cost into the calculations. Our AHP framework presents a different concept of driving risk factors by differentiating between driver states and driver behavior criteria and by adding an environmental criterion. The sub-criteria for driver states and behaviors are based on different methods from those in the previously mentioned studies.

This study aims to introduce a novel SMCRisk. This model is an integral part of our research related to a new concept of holistic risk assessment introduced by Carrodano [37], called HoloRisk, and defined as *“HoloRisk is based on all aspects and events related to a person's life. The underlying concept is that all actions, events, and states of the environment have an impact on the risk level in every aspect of the person's life and environment. How things are correlated and what is the impact are the central questions of HoloRisk”.* The novel concept of HoloRisk contemplates the elements that surpass an individual's direct influence. SMCRisk served as a foundation for risk assessment in this study.

To summarize, the main contributions of the SMCRisk model are as follows:

1) The aforementioned risk-analysis MCDM-related studies used surveys to determine a pairwise comparison matrix. The semi-quantitative method lowers the subjectivity of expert opinions for the pairwise comparison matrix by using objective sources such as statistics alongside expert judgment. The majority of the literature reviewed on road risk analysis using MCDM is based on surveys. To the best of our knowledge, this is the first time statistics have been combined with expert judgment for PCM in the field of risk analysis.
2) The weighting integrates a severity cost factor, implying that risk intensity is considered. The integration of a cost factor is a novel method in risk analysis using MCDM.

The remainder of this paper is organized as follows: The first section covers the mathematical foundation of the AHP as well as our proposed approach and methodology. The second section focuses on a case study that examines driving risks in the USA in 2019, followed by the identification of risk factors and the modeling process. In the third section, we discuss the results and conclude the paper.

## 2 Mathematical background

Before introducing our innovative method, we first outline the mathematical foundations underlying our proposal. This section comprises three subsections. The first subsection explains the AHP, followed by the second subsection, which covers the pairwise comparison matrix and the concept of consistency. The third subsection describes the proposed approach and methodology.

### 2.1 Analytic hierarchy process (AHP)

Thomas Saaty conceived the AHP in the 1970s as a highly structured technique to analyze complex decision-making while minimizing bias. Drawing from psychological and mathematical foundations, a key element of the AHP is the *“pairwise comparison matrix,”* which requires the comparative judgment of various components of the decision. This results in a more granular assessment, while considering all relevant criteria in a hierarchical form.

According to Saaty [38], psychological underpinning is derived from cognitive psychologist Blumenthal [39], who proposed the concept of making judgments through a comparison process. Saaty [38] argued that, regardless of the nature of a decision–whether tangible or intangible–its interpretation remains subjective. AHP offers a suitable method for examining all aspects of the decision-making process, relying primarily on expert opinions to evaluate various components in relation to one another.

The mathematical framework of the AHP was built through a series of steps [40,42]. These encompass initially setting up the problem in a hierarchical structure, carrying out pairwise evaluations, constructing a PCM, checking consistency, and finally calculating the weights based on the findings from the preceding stages. The steps are detailed below.

1. **Problem Structuring**: Outline the problem and determine its objectives. Identify the criteria and sub-criteria. Organizing these elements into a hierarchical structure.
2. **Pairwise Comparisons Matrix**: Performs pairwise comparisons for criteria and sub-criteria. Each element in a given level of hierarchy is compared in pairs to determine which element is more important or has a greater influence than the goal or criterion above it in the hierarchy. Comparisons were typically made using a scale ranging from one (equal importance) to nine (extreme importance).
3. **Weighting**: The results of the pairwise comparisons were used to calculate the weights for each element in the hierarchy. This is typically performed using eigenvector calculations.
4. **Validation**: Validation of the consistency of the PCM. This step is crucial because it verifies whether the comparisons are logically consistent. If the consistency ratio is too high (greater than 0.1), the comparisons may be inconsistent.
5. **Synthesis:** Aggregate the weights of the sub-criteria across all the main criteria to calculate the overall weight of each sub-criterion. Subsequently, a ranking is established based on these weights.

### 2.2 Pairwise comparison matrix (PCM)

The following explanations are based on Saaty [[40], [41], [42]]: The core concept of AHP is a hierarchical model that divides the problem into different aspects, including criteria and sub-criteria. This hierarchical structure enables a comprehensive understanding of the problem and its components.

Once the hierarchical model is established, experts evaluate various elements within the hierarchy using a numerical scale [Table 1]. This process involves assigning relative importance values to each element by conducting pairwise comparisons. The experts assess and compare the elements in pairs, enabling them to systematically determine the significance of each element in relation to others within the same level of the hierarchy. These judgments aid determine the importance intensities, which are subsequently used to create a PCM.

**Table 1:** Numerical scale of Saaty [40].

Intensity of Importance	Definition	Explanation of the fundamental values
1	Equal importance	Two elements contribute equally to the objective
3	Moderate importance	Experience and judgment slightly favor one element over another
5	Strong importance	Experience and judgment strongly favor one activity over another
7	Very strong importance	An element is favored very strongly over another
9	Extreme importance	The evidence favoring one element over another is of the highest possible order of affirmation.

1 2, 4, 6, and 8 are considered as “intermediate values” by Saaty [38,40]. These values are used when the choice between two fundamental values (1, 3, 5, 7, or 9) requires a compromise.

The PCM (Equation (1)) is a square matrix that represents the relative importance of each alternative with respect to all others. Each entry in matrix (*a*_*ij*_) corresponds to the ratio of the importance intensities (*w*_i_) of alternatives i and j (i.e., *a*_*ij*_ = *w*_*i*_*/w*_*j*_). The diagonal elements of the matrix are always equal to one because the alternatives are compared.

Using the pairwise comparison method, each alternative was assigned an importance intensity. We then obtain a PCM, as follows (Equation (1)):

(1) $$Pairwise\;comparison\;matrix\;A=\left[\begin{array}{ccc} w_{1}/w_{1} & \cdots & w_{1}/w_{n}\\ \vdots & \ddots & \vdots \\ w_{n}/w_{1} & \cdots & w_{n}/w_{n} \end{array}\right]$$

where *w* (*w*_*1*_*, …, w*_*n*_) represent the *“weights”* allocated to each alternative (or sub-criteria).

Matrix *A* ($A=a_{ij})$ has two properties: positive entries (Equation (2)) and reciprocal properties (Equation (3)). Matrix A is referred to as the reciprocal matrix.

(2) $$a_{ij}>0$$

(3) $$a_{ji}=\frac{1}{a_{ij}}\forall i,j$$

Considering the geometric interpretation of the linear systems, the calculation can be performed using the following (Equation (4)):

(4) Aw=nw

where *n* represents an eigenvalue of *A* and *w* represents the associated eigenvector, which is detailed in the following equation (Equation (5)):

(5) $$Aw=\left[\begin{array}{ccc} w_{1}/w_{1} & \cdots & w_{1}/w_{n}\\ \vdots & \ddots & \vdots \\ w_{n}/w_{1} & \cdots & w_{n}/w_{n} \end{array}\right]\left[\begin{array}{c} w_{1}\\ \vdots \\ w_{n} \end{array}\right]=n\left[\begin{array}{c} w_{1}\\ \vdots \\ w_{n} \end{array}\right]=nw$$

For consistency, a linear system should have at least one solution; if the matrix has no solution, the system is inconsistent.

Matrix A is consistent if it satisfies the following condition (Equation (6)):

(6) $$a_{jk}=\frac{a_{ik}}{a_{ij}}\;i,j,k=1,\ldots ,n$$

Given that the PCM is defined by expert judgment, it is assumed that the values *w* might be imprecise. Therefore, the matrix may not be consistent, and *n* is not an eigenvalue of Equation (4).

However, considering the inconsistency of the expert judgment matrix and assuming that the equation to be solved has a solution, we replaced *n* with the lambda maximum as the eigenvalue [4].

The new equation is as follows (Equation (7)):

(7) $$Aw=\lambda_{\max}\;w$$

where per Saaty [40] theorems, $\lambda_{\max}\geq n$ and the matrix is consistent if and only if $\lambda_{\max}=n$. We assume that Equation (4) is a special case of general Equation (7).

According to Saaty [40] and Vargas [43], the inconsistency of a matrix must be less than 10 % to allow the eigenvector to follow a Dirichlet distribution. It indicates that the following consistency ratio, CR (Equation (8)) must be less than 0.10.

(8) $$CR=\frac{CI}{RI}$$

where the RI represents the average random consistency index [Table 2] and the consistency index (CI) is computed as follows (Equation (9)):

(9) $$CI=\frac{\lambda_{\max}-n}{n-1}$$

where $\lambda_{\max}$ represents the maximum eigenvalue and n represents the matrix order or the number of criteria.

**Table 2:** Random index (RI).

n	1	2	3	4	5	6	7	8
RI	0	0	0.58	0.90	1.12	1.24	1.32	1.41

## 3 Our proposal: A semi-quantitative multi-criteria model (SMCRisk)

Because the statistics available in the context of mobility risk are not entirely accessible or may omit certain information relevant to our hierarchical model, we propose a hybrid approach that integrates a qualitative method (expert judgment) with a quantitative one.

Statistical data provide objective evidence for risk assessment. However, constraints can emerge from privacy issues, the omission of crucial information for analysis, or the application of different methodologies. Consequently, statistics may differ in source, structure, and content, requiring a final evaluation by experts.

The growth of IoT will enhance the availability of information that can be incorporated into the hierarchical model, facilitating a deeper comprehension of specific risk factors. The study proposed below lays the foundation for a hierarchical method, which could be further refined in the future as more data from diverse sources become accessible.

In our risk assessment method, we allocated a cost to account for and amplify the consequences of risk factors. The PCM was developed by considering the statistically based probability for a one-year period, multiplied by a cost representing severity. Severity was determined by the magnitude of human consequences, such as fatalities or injuries, whereas non-human consequences include only property damage. After constructing the matrix, we used the eigenvalue method (EM) and evaluated the consistency ratio (CR) in accordance with previously outlined methods.

**Fig. 2:**
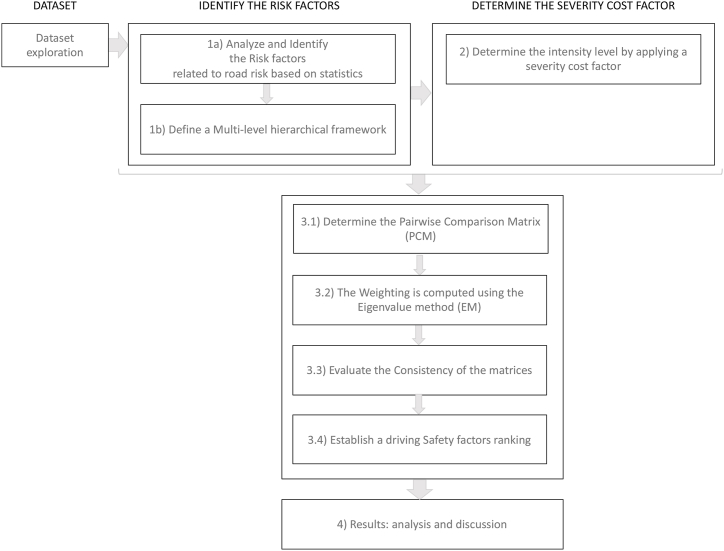
Methodology of the SMCRisk model.

The methodology of the SMCRisk model is illustrated in Fig. 2 and described below:

Step 1 We begin with an in-depth analysis of the statistical aspects of driver risk by identifying risk factors, which are then categorized into criteria and sub-criteria. The criteria represent the primary categories to which the subcriteria are allocated. We first explore the dataset to analyze and identify risk factors related to driving risks using official statistics. After analyzing the dataset, we establish a multi-level hierarchical framework.

Step 2 Determine the severity level by calculating the normalized cost factor. At this stage, criteria and sub-criteria are defined, and we prepare the data for a PCM based on a semi-qualitative method for the criteria and a quantitative method for the sub-criteria, combining the severity cost factor with statistical crash accidents.

Step 3.1 We establish the PCM.

Step 3.2 We assess the weights of the criteria and sub-criteria using the eigenvalue method (EM).

Step 3.3 Evaluate the consistency of the matrices.

Step 3.4 We provide a driving safety factor ranking.

Step 4 We analyze and discuss the result

The following section introduces a case study focused on driving risks in the US.

## 4 Case study: US driving risks

### 4.1 Dataset exploration

The case study was based on real data. The dataset for this study was sourced from the *National Highway Traffic Safety Administration* (NHTSA), which is available on the open-source NHTSA platform (https://www.nhtsa.gov/). The retrieved crash report dataset refers to accidents with fatalities in all US states from January 1, 2019, to December 31, 2019.

**Fig. 3:**
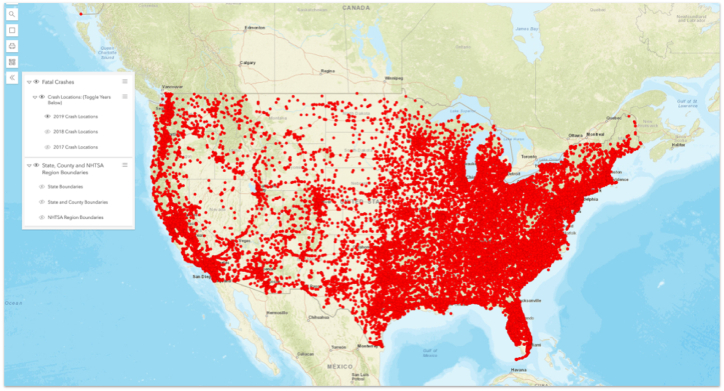
Fatal Crash locations in 2019 (Source: NHTSA).

The dataset consists of 33,244 rows (or fatal crashes) and 81 variables. Some of the major variables include date, state, daytime or nighttime, weather conditions, rural or urban setting, behavior (speeding), rollover, road types, persons involved in the crash (young, for instance), age, and many other variables. The dataset was filtered only to include fatal crashes because there were more available variables for nonfatal crashes. The total 33,244 rows (fatal crashes) correspond to the NHTSA Traffic Safety Facts 2019 Report (https://crashstats.nhtsa.dot.gov/Api/Public/ViewPublication/813141). We extracted other crash reports for the same period and crash types from the NHTSA website to complete and analyze the case study. The 2019 fatal crash locations are shown in the following map (Fig. 3).

### 4.2 Risk factors identification and the multi-level hierarchical framework

**Fig. 4:**
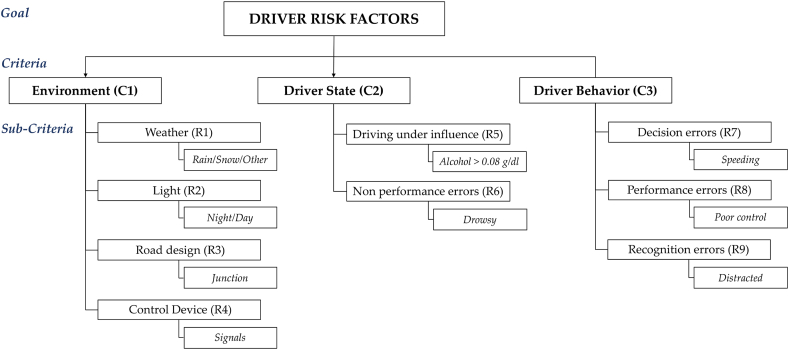
Multi-level hierarchical framework for driving risks factors.

The multi-level framework for driving risk factors is shown in Fig. 4, and is based on our analysis of fatal crashes that occurred in 2019.

We identified three primary criteria: the *environment* (C1), *driver state* (C2), and *driver Behavior* (C3). These criteria were based on the main factor categories implied in the risk levels related to fatal crashes. Each criterion is expressed using several main sub-criteria.

The *environment* criteria is subdivided into four main sub-criteria: *weather* (R1), *light* (R2), *road design* (R3), and *control devices* (R4). *Driving under the influence* (R5) and *non-performance errors* (R6) are the two sub-criteria of the *driver state*. *Driver behavior* is related to *decision errors* (R7), *performance errors* (R8), and *recognition errors* (R9).

To categorize driver behavior, we analyzed the driving factors based on Parker et al. in 1995 [45] and those proposed by NHTSA [44]. Parker et al. [45] identified three behavioral factors: *errors*, which involve errors while driving; *violations errors* associated with drivers who violate driving practices; and *lapses errors*, which are minor errors stemming from lack of attention. By contrast, NHTSA categorizations [44] are based on statistics and are classified into four main factors: *recognition errors, decision errors, performance errors,* and *non-performance errors*. *Recognition errors* include driver inattention or distraction and inadequate surveillance. The *decision errors* imply speeding, false assumptions about others ‘actions, and illegal driving behavior. The main *performance errors* are overcompensation and poor directional control. The last type, *non-performance errors*, is primarily due to the driver's state, such as drowsy drivers.

Considering some of the main driver behaviors (Table 3), we defined them based on NHTSA [44] (D = decision errors, R = recognition errors, P = performance errors, N = non-performance errors) and Parker et al. [45] (V = violation errors, E = errors, L = lapse errors). We distinguished driver behaviors and driver states as sub-criteria. We have added the state (S) for driving under influence (DUI). The state sub-criteria also include non-performance errors such as drowsy drivers.

**Table 3:** Driver behaviors factors and NHTSA-Parker comparison.

Risk Factors	NHTSA	Parker et al.
Speeding	D	V
DUI	S	V
Fail to yield	D	V
Reckless driving	D	V
Wrong lane	R	E
Careless driving	R	E
Distracted driver	R	E
Traffic signs, lights	R	E
Swerving (environment)	P	E
Overcorrecting	P	E
Poor vision	R	E
Drowsy	N	L
Wrong way	R	L
Wrong turn	R	L

Our AHP framework uses an adapted version of the NHTSA driver behavior, which involves extracting driver states, specifically nonperformance errors.

The subsequent sections focus on constructing a quantitative PCM and weighting process using the eigenvalue method.

### 4.3 Determine the severity cost

In this section, we discuss the methods used to establish pairwise matrices in our method. For the first level, which involved categories, we used a qualitative method that combined statistics with expert judgment. For the second level, which refers to the sub-criteria, we use a quantitative method that considers statistics along with a severity cost factor. This process is described in detail as follows.

The *Traffic Safety Facts 2019 Reports* of the NHTSA present statistics related to “crash severity”, distinguishing fatalities, injuries, and property damage-only crashes. Based on these statistics, we developed a severity cost factor (Table 4) that considers the severity of the consequences of a crash.

**Table 4:** Severity cost factor.

Types	Crashes consequences	Cost factor	Crashes	Crashes x cost	Normalized costs factor
Fatal	Fatal	332	33′244	11′037′008	0.470
Major	Injury	4	1′916′000	7′664′000	0.326
Minor	Property	1	4′806′000	4′806′000	0.204

Table 4 lists the severity of the crashes mentioned as fatal, major, and minor consequences. We allocated a cost of 332 for fatal outcomes, 4 for major consequences or injuries, and 1 for minor consequences related to properties only. If this information was available, we would have considered a more nuanced method, such as injury with or without consequences. Based on the number of crashes multiplied by cost and normalized, we obtain the normalized severity factor.

The cost factors are based on the US costs for crash deaths, body injuries, and property damage. The 2020 Insurance Information Institute reports mentions the average settlements of property damage and bodily injuries. These amounts were $4711 and $20235 (https://www.forbes.com/advisor/legal/auto-accident/typical-car-settlement-amounts/). The 2018 cost of crash deaths was $55 billion for medical and work loss costs in the US (https://www.cdc.gov/transportationsafety/statecosts/index.html) for 38000 people, reported to 2019 statistics with approximately 36000 deaths, it implies a cost of $52 billion. By dividing by the number of crashes, the average cost per crash was $1,565,000.

The cost factor is proportional to the cost of a property damage crash and is equal to 1. The injuries were calculated as $20235/$4711, which is 4. Crashes involving injured people are four times more expensive than crashes without injuries (property only). The fatal crashes were calculated as $1,565,000/$4711 which is equal to 332. This implies that fatal crashes are 332 times more expensive than crashes involving property damage only.

Note that this severity cost factor is an approximation that may change annually. The concept is to consider the approximate severity level in the global risk calculation. Using the NHTSA 2019 statistics, we computed a ratio considering the severity factor for the environment, driver state, and driver behavior subcriteria, as shown in Table 5.

**Table 5:** Quantitative method including severity cost factor for sub-criteria.

Environment	R1	R2	R3	R4
Fatal	1.4 %	15.1 %	13.6 %	7.8 %
Injury	4.1 %	4.4 %	17.5 %	11.3 %
Property	2.9 %	3.1 %	9.7 %	6.2 %
*Sum*	*8.4 %*	*22.6 %*	*40.8 %*	*25.3 %*

Driver State	R5	R6
Fatal	14.3 %	1.1 %
Injury	2.9 %	0.6 %
Property	1.0 %	0.3 %
*Sum*	*18.2 %*	*2.0 %*

Driver Behavior	R7	R8	R9
Fatal	12.2 %	7.4 %	4.1 %
Injury	3.9 %	1.2 %	4.9 %
Property	1.8 %	0.2 %	3.0 %
*Sum*	*17.9 %*	*8.8 %*	*12.0 %*

### 4.4 Criteria and sub-criteria scale based on the pairwise comparison matrix

The previous qualitative and quantitative methods are represented as a PCM (Table 6). The criteria-level square matrix was a 3 × 3 matrix. The sub-criteria matrices have respectively 4 × 4 for the environment, 2 × 2 for the driver state, and 3 × 3 *n* order for driver behavior. The scale of relative importance for the qualitative method refers to Saaty [40], and includes levels ranging from one (equal importance) to nine (extreme importance).

**Table 6:** Pairwise comparison matrices for criteria and sub-criteria.

Criteria	C1	C2	C3	
C1	1	1/4	1/5	
C2	4	1	1	
C3	5	1	1	
Sub-Criteria	R1	R2	R3	R4
R1	1	1/3	1/5	1/3
R2	3	1	1/2	1
R3	5	2	1	2
R4	3	1	1/2	1
Sub-Criteria	R5	R6		
R5	1	9		
R6	1/9	1		
Sub-Criteria	R7	R8	R9	
R7	1	2	2	
R8	1/2	1	1/2	
R9	1/2	2	1	

### 4.5 Weighting process using the eigenvalue method (EM)

At this stage, we proceed with a weighting process using the eigenvalue method (Table 7). We first normalized the matrices, followed by a weight calculation.

**Table 7:** Normalized matrices.

Criteria	C1	C2	C3	
C1	0.100	0.112	0.090	
C2	0.400	0.444	0.455	
C3	0.500	0.444	0.455	
Sum	1.000	1.000	1.000	
Sub-Criteria	R1	R2	R3	R4
R1	0.083	0.076	0.091	0.076
R2	0.250	0.231	0.227	0.231
R3	0.417	0.462	0.455	0.462
R4	0.250	0.231	0.227	0.231
Sum	1.000	1.000	1.000	1.000
Sub-Criteria	R5	R6		
R5	0.900	0.900		
R6	0.100	0.100		
Sub-Criteria	R7	R8	R9	
R7	0.500	0.400	0.571	
R8	0.250	0.200	0.143	
R9	0.250	0.400	0.286	
Sum	1.000	1.000	1.000	

After normalizing the previous matrices, we calculate the weights (Table 8).

**Table 8:** Criteria and sub-criteria weighting.

Criteria	Weighting	Sub-criteria	Weighting
C1	0.101	R1	0.082
C2	0.433	R2	0.235
C3	0.466	R3	0.448
		R4	0.235
		R5	0.900
		R6	0.100
		R7	0.493
		R8	0.196
		R9	0.311

### 4.6 Evaluation of the matrices

The consistency of the matrices was evaluated by computing the consistency ratio (CR). If CR was <0.10, the matrix was considered to be consistent (Table 9).

**Table 9:** Lambda max, consistency index (CI), random consistency index (RI), and consistency rate (CR).

Levels	λmax	CI	RI	CR
Criteria	3.0055	0.0028	0.58	0.0047
Sub-Criteria Environment	4.0042	0.0014	0.90	0.0016
Sub-Criteria Driver behavior	3.0537	0.0269	0.58	0.0463

The consistency rate (CR) was calculated. The matrix structure is respectively 3 × 3 (n = 3) for the criteria, 4 × 4 (n = 4) for the environment, and 3 × 3 (n = 3) for the driver behavior.

Criteria:

$$CR=\frac{CI}{RI}=\frac{\left(\lambda \;\max-n\right)/\left(n-1\right)}{RI}=\frac{\left(3.0055-3\right)/\left(3-1\right)}{0.58}=0.004741$$

(CR<0.10)

Sub-criteria (Environment):

$$CR=\frac{CI}{RI}=\frac{\left(\lambda \;\max-n\right)/\left(n-1\right)}{RI}=\frac{\left(4.0042-4\right)/\left(4-1\right)}{0.90}=0.001555$$

(CR<0.10)

Sub-criteria (Driver behavior):

$$CR=\frac{CI}{RI}=\frac{\left(\lambda \;\max-n\right)/\left(n-1\right)}{RI}=\frac{\left(3.0537-3\right)/\left(3-1\right)}{0.58}=0.046293$$

(CR<0.10)

All criteria and sub-criteria matrices have a consistency rate lower than 0.10 indicating a high level of consistency.

### 4.7 Driving safety factors ranking

**Fig. 5:**
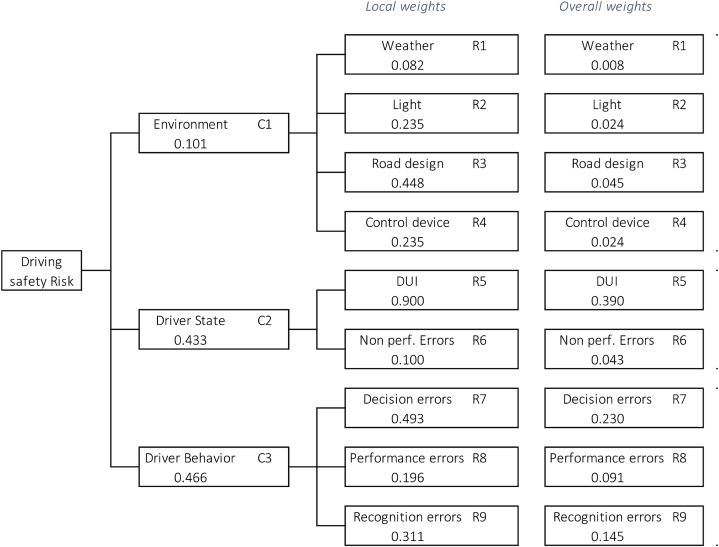
Weighted criteria and sub-criteria (local weights) and overall weights calculations.

Fig. 5 shows the local and overall weights of the criteria and sub-criteria.

The driving safety factors are ranked in the following figure (Table 10).

**Table 10:** Driving safety factors ranking (*same ranking level).

	Criteria	Weights	Ranking
C1	Environment	0.101	3
C2	Driver State	0.433	2
C3	Driver Behavior	0.466	1

	Criteria	Weights	in %	Ranking
R1	Weather	0.008	1 %	9
R2	Light	0.024	2 %	7*
R3	Road design	0.045	5 %	5
R4	Control device	0.024	2 %	7*
R5	DUI	0.390	39 %	1
R6	Non perform. Errors	0.043	4 %	6
R7	Decision errors	0.230	23 %	2
R8	Performance errors	0.091	9 %	4
R9	Recognition errors	0.145	14 %	3

## 5 Comparisons, results analysis and discussion

In this section, we propose a comparison of SMCRisk with other subjective models, such as BWM and FUCOM, and then analyze and discuss the results.

### 5.1 Comparisons

**Fig. 6:**
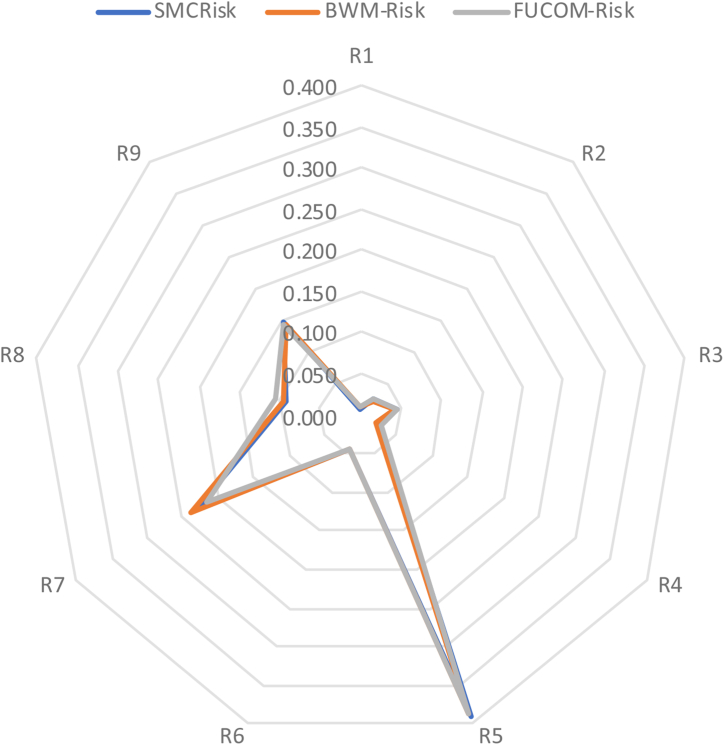
Graphical representation: Comparisons SMCRisk-BWM-Risk-FUCOM-Risk.

A comparison was made with two other subjective models, BWM and FUCOM. Both were modified to include the risk factors in the process; therefore, we refer to them as BWM-Risk and FUCOM-Risk, respectively. The novelty of the proposed model lies in the PCM process. The method we used is not based on surveys, which result in a high degree of subjectivity; instead, it uses objective sources and expert knowledge. The determination of risk factors related to risk severity was aggregated during the PCM process. We then used the previously mentioned AHP method. The choice of the AHP lies in its simplicity and multi-level characteristics, which are perfectly suited for risk analysis. Therefore, we proposed modifying the BWM and FUCOM methods to integrate the risk factors used in the previous example. The results are presented as follows:

The graphical representation of Table 11 is shown hereafter (Fig. 6).

**Table 11:** Comparisons SMCRisk, BWM-Risk, and FUCOM-Risk weights and ranking (*similar level).

Methods	R1	R2	R3	R4	R5	R6	R7	R8	R9
SMCRisk	0.008	0.024	0.045	0.024	0.390	0.043	0.230	0.091	0.145
*Rank*	*9*	7*	*5*	7*	*1*	*6*	*2*	*4*	*3*
BWM-Risk	0.008	0.022	0.043	0.022	0.386	0.043	0.238	0.095	0.143
*Rank*	*9*	7*	*6*	7*	*1*	*5*	*2*	*4*	*3*
FUCOM-Risk	0.009	0.024	0.044	0.027	0.387	0.043	0.215	0.106	0.144
*Rank*	*9*	*8*	*5*	*7*	*1*	*6*	*2*	*4*	*3*

The Table 11 shows a comparison of the three methods (SMCRisk, BWM-Risk, and FUCOM-Risk) for nine criteria (R1–R9). The ranking values reflect similarities, and the weight coefficient values show little difference. PCM integrates risk factors and semi-quantitative processes. These results demonstrate that the proposed process can be applied to other MCDM methods. In the pairwise comparison process, each method has its advantages and disadvantages.

BWM-Risk requires the selection of the best and worst criteria before actually knowing them, increasing the level of subjectivity. One of the purposes of the proposed model is to increase objectivity. However, the BWM method is not ideal for this purpose. FUCOM has the advantage of directly integrating statistical data, unlike AHP, which requires an intermediate step in which data are structured into a matrix, referencing Saaty's numerical scale ranging from 1 to 9 [40]. This process results in an approximation. However, this step integrates the final expert evaluation. The AHP method has a distinct advantage because it does not require prior knowledge of preferences as per FUCOM; this information is unknown at that stage, as provided by objective sources, and then finalized with expert judgment.

This comparison reinforces our method selection, SMCRisk, based on the AHP. The use of BWM is not appropriate at all, and FUCOM requires some modifications in the early process stage but may be an interesting alternative. The results of the SMCRisk application are discussed in the following section.

### 5.2 Results analysis and discussion

The proposed method yields satisfactory results. The matrices have been shown to be consistent. Moreover, the results align with real-life situations as they are partially based on statistical analysis. This study highlighted the significant risk associated with driver behavior and the comparatively low impact of environmental factors.

**Fig. 7:**
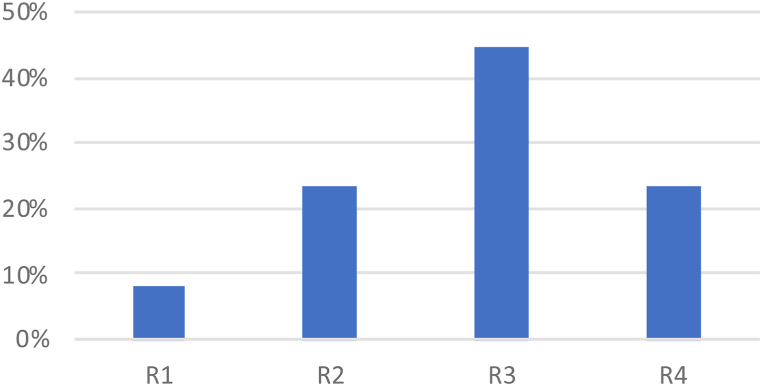
Environment sub-criteria (local weights) in percent.

At a more granular level, the environment sub-criteria accounted for 8 % for weather, 24 % for light, 44 % for road design, and 24 % for the control device (Fig. 7). The road design was identified as the most significant risk factor.

The driver's state was markedly influenced by driving under the influence of alcohol consumption, contributing to 90 %. This was the most significant overall risk factor.

Alcohol consumption can significantly impair motor skills and slow reaction times. The driver's ability to make quick decisions on the road or recognize potential hazards is also compromised. Thus, effects of alcohol consumption on visual capabilities may impair judgment, making it difficult for drivers to estimate distances and react to changes in light of movements. It may also instill false confidence in individuals, leading to risk-taking behaviors.

**Fig. 8:**
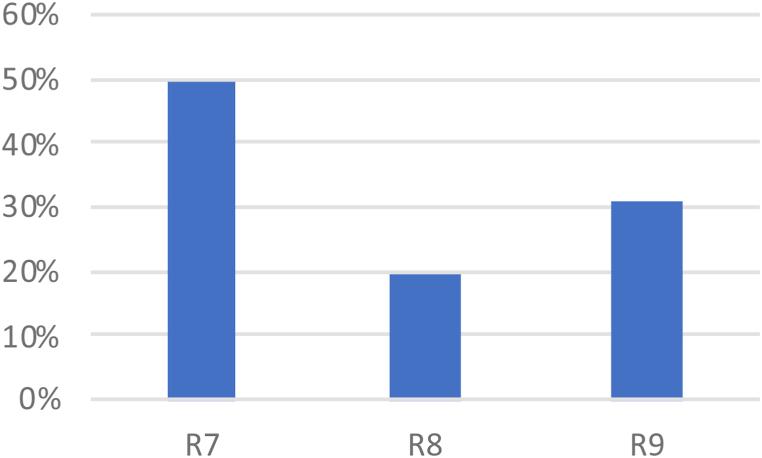
Driver behavior sub-criteria (local weights) in percent.

The sub-criteria of driver behavior and the proportions of decision, performance, and recognition errors were 49%, 20%, and 31%, respectively, as shown in Fig. 8.

The decision error associated with speeding was the most significant. This was followed by a recognition error linked to distracted drivers. Speeding is often the result of a conscious decision by a driver. Drivers may accelerate for several reasons, such as impatience, running late, or overconfidence in their driving skills. However, higher speeds reduce the driver's ability to steer safely around curves or objects on the roadway, extend the distance necessary to stop the vehicle, and increase the distance a vehicle travels while reacting to dangerous situations. These factors result in an increase in the probability of crashing, which is a significant risk factor.

Another important risk factor related to distracted drivers is the use of mobile phones, eating, or radio adjustments. These may divert the driver's attention from the road and result in the driver missing critical events, objects, and cues or losing control of the vehicle, leading to crashes. This factor has become a serious concern because of increasing reliance on digital devices.

Considering the overall weights, the study revealed that driving under the influence, decision errors (e.g., speeding) and recognition errors (e.g., distracted driving) were the top three risk factors for driving safety. These factors closely correspond to those mentioned by the WHO and NHTSA, as noted in the introduction. The most significant risks identified included speeding, driving under influence, and distracted driving.

Our study suggests that driver behavior and state are the most significant risk factors. There are several possible explanations for these findings. We suggest some general considerations, considering that these explanations might vary depending on local conditions.

Modern vehicles are equipped with advanced safety features such as ABS, traction control, stability control, and collision detection systems. Considerable improvements in road design, signage, and lighting can reduce the number of accidents caused by environmental factors. Thus, reducing the impact of other factors induces driver behavior to become more prominent.

From another perspective, certain measures have been implemented to increase awareness of the risk factors associated with driver state and behavior. Over the years, there has been an increased emphasis on driver education, awareness of driving-safety rules, and the dangers of distracted or impaired driving. This increased awareness may result in safer driving habits. Law enforcement agencies have become stricter regarding road-safety regulations. Penalties for traffic violations, particularly for driving under influence, have become more severe. The increase in the use of public transportation and ride-sharing services might have reduced the number of inexperienced or impaired drivers on the road. While these initiatives contribute to the declining trend in accidents, their limited effect is likely attributable to the extended period required to yield significant results for the actual accident rate.

Indirect measures such as advancements in data collection and analysis have fostered an improved comprehension of road accidents and their contributing factors. This enhanced understanding will facilitate the implementation of more effective interventions and policies.

Potential obstacles to reducing accident rates may include subjective elements such as risk perception and risk culture. Psychological aspects and individual personality traits may also play a role in encouraging riskier behaviors.

## 6 Conclusion

MCDM methods for risk analysis demonstrate the use of surveys in the PCM process. This method has a high level of subjectivity, considering that risk might be perceived differently by each individual. Many biases can emerge during the judgment process, resulting in the incorrect consideration of risk factors. Thus, risks must be identified in a more objective manner. We propose using objective information, such as statistics, to build the PCM. Because this information originates from various sources, it may have varying structures and methodologies. We assume that a final expert judgment is necessary to harmonize the pairwise comparison process. The other novelty of the SMCRisk proposal is that it considers risk intensity by applying a risk factor integrated into the weights. To the best of our knowledge, this integration is completely new, as is the use of objective sources combined with expert judgment. For our model, we defined the criteria and sub-criteria based on the NHTSA and not Parker's approach, as proposed in other studies. The NHTSA proposal shows a wider consideration of driving behaviors than Parker's proposal, which focuses on the non-consideration of road regulations.

Applying SMCRisk to real-world scenarios affirms its veracity, with the outcomes mirroring the established analysis in the domain.

However, our model does not incorporate technical aspects such as engine issues or vehicle materials owing to data scarcity and their limited influence on driving safety. Thus, we corroborate that the previous assessment, emphasizing a slight reduction in fatal accidents from 2015 onwards, is attributable to driver behavior.

In the future, as additional data becomes accessible, we propose the inclusion of more risk factors, such as the incorporation of autonomous or semi-autonomous vehicles into road traffic. These emerging technologies may introduce new risks, including cybersecurity vulnerabilities or the requirement for drivers to remain alert in certain situations. Other evolving risk factors, such as those associated with low-altitude unmanned aerial vehicles, can also be considered.

Although the model is relevant and accurate, its primary limitation stems from a lack of sufficient data. This includes a cost factor, which can be refined and calculated more accurately with access to more information. Similarly, as data availability increases, the granularity of a study can be improved. SMCRisk was designed specifically for risk analysis, considering the intensity of the risk. This may be applied to safety fields considering the intensity level of risk but may not be appropriate for all domains, such as product selection.

We introduce a novel semi-quantitative technique using the AHP to evaluate risk within road traffic contexts. SMCRisk presents different characteristics: objectivity is significantly improved by decreasing the individual bias from expert judgment related to risk perception, and risk severity is integrated into the weights to perform an accurate ranking. The method used was simple and appropriate, with an interesting multi-level structure for the holistic and heterogeneous sources required for risk analysis.

The implications of the SMCRisk are theoretical and practical. From a theoretical perspective, the proposed novel method considerably modifies the PCM method, which is based only on a subjective approach, by introducing semi-quantitative aspects. Another theoretical implication relates to the integration of risk intensity into the weighting process.

The practical implications refer to the use of this method. Ranking the risk factors in the field of risk analysis is an important task. In the context of road traffic, this may also result in a better understanding of the most important risk factors and the definition of more targeted prevention measures to reduce road accidents.

This groundbreaking SMCRisk forms the basis for future progress by embracing emerging risks. Our proposed multi-level method facilitates the handling of the heterogeneous data required to analyze risk factors. In the future, this method can be expanded to directly integrate data from the IoT, enabling its implementation in a more extensive computational framework.

This study is part of our broader research on HRA.

## Funding

This research did not receive any specific grants from funding agencies in the public, commercial, or not-for-profit sectors.

## Data availability statement

Data included in article/supp. Material/referenced in article.

## CRediT authorship contribution statement

**Cinzia Carrodano:** Conceptualization, Data curation, Formal analysis, Investigation, Methodology, Visualization, Writing – original draft.

## Declaration of competing interest

The authors declare that they have no known competing financial interests or personal relationships that could have appeared to influence the work reported in this paper.
